# Icaritin inhibits neuroinflammation in a rat cerebral ischemia model by regulating microglial polarization through the GPER–ERK–NF-κB signaling pathway

**DOI:** 10.1186/s10020-022-00573-7

**Published:** 2022-11-26

**Authors:** Zining Yu, Guangjun Su, Limei Zhang, Gaigai Liu, Yonggang Zhou, Shicai Fang, Qian Zhang, Tianyun Wang, Cheng Huang, Zhihua Huang, Liangdong Li

**Affiliations:** 1 First Affiliated Hospital of Gannan Medical University, Ganzhou, 341000 China; 2 Key Laboratory of Prevention and Treatment of Cardiovascular and Cerebrovascular Diseases of Ministry of Education, Gannan Medical University, Ganzhou, 341000 China; 3 Ganzhou Key Laboratory of Neuroinflammation Research, Gannan Medical University, Ganzhou, 341000 China; 4 Institute for Medical Sciences of Pain, Department of Physiology, School of Basic Medical Sciences, Gannan Medical University, Ganzhou, 341000 China; 5 School of Basic Medicine Sciences, Gannan Medical University, Ganzhou, 341000 China; 6 Graduate School, Gannan Medical University, Ganzhou, 341000 China

**Keywords:** Cerebral ischemia/reperfusion injury, Icaritin, Microglia, GPER, ERK, NF-κB

## Abstract

**Background:**

Activated microglia play a key role in initiating the inflammatory cascade following ischemic stroke and exert proinflammatory or anti-inflammatory effects, depending on whether they are polarized toward the M1 or M2 phenotype. The present study investigated the regulatory effect of icaritin (ICT) on microglial polarization in rats after cerebral ischemia/reperfusion injury (CI/RI) and explored the possible anti-inflammatory mechanisms of ICT.

**Methods:**

A rat model of transient middle cerebral artery occlusion (tMCAO) was established. Following treatment with ICT, a G protein-coupled estrogen receptor (GPER) inhibitor or an extracellular signal-regulated kinase (ERK) inhibitor, the Garcia scale and rotarod test were used to assess neurological and locomotor function. 2,3,5-Triphenyltetrazolium chloride (TTC) and Fluoro-Jade C (FJC) staining were used to evaluate the infarct volume and neuronal death. The levels of inflammatory factors in the ischemic penumbra were evaluated using enzyme-linked immunosorbent assays (ELISAs). In addition, western blotting, immunofluorescence staining and quantitative PCR (qPCR) were performed to measure the expression levels of markers of different microglial phenotypes and proteins related to the GPER–ERK–nuclear factor kappa B (NF-κB) signaling pathway.

**Results:**

ICT treatment significantly decreased the cerebral infarct volume, brain water content and fluorescence intensity of FJC; improved the Garcia score; increased the latency to fall and rotation speed in the rotarod test; decreased the levels of interleukin-1 beta (IL-1β), tumor necrosis factor-alpha (TNF-α), Iba1, CD40, CD68 and p-P65-NF-κB; and increased the levels of CD206 and p-ERK. U0126 (an inhibitor of ERK) and G15 (a selective antagonist of GPER) antagonized these effects.

**Conclusions:**

These findings indicate that ICT plays roles in inhibiting the inflammatory response and achieving neuroprotection by regulating GPER–ERK–NF-κB signaling and then inhibiting microglial activation and M1 polarization while promoting M2 polarization, which provides a new therapeutic for against cerebral ischemic stroke.

**Graphical Abstract:**

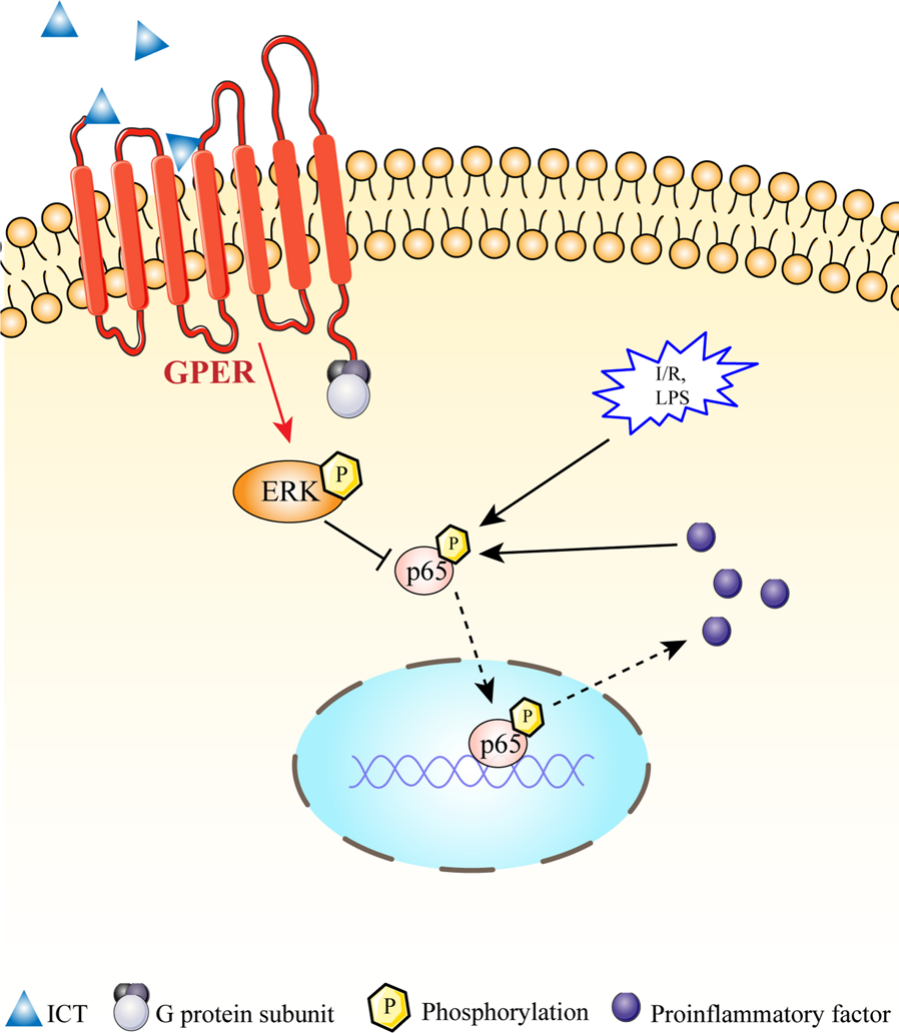

## Introduction

Ischemic stroke is a disease with high mortality and disability rates worldwide. It is mainly caused by arterial embolism, microangiopathy or macroangiopathy and induces reperfusion injury when the blood vessels are recanalized (Bang et al. 2016; Meloux et al. 2018). The pathogenesis of brain tissue damage caused by cerebral ischemia/reperfusion injury (CI/RI) is very complex and involves oxidative stress, excitotoxicity, the release of endogenous substances (such as chemokines, nitric oxide, and reactive oxygen species), inflammation, apoptosis and other processes (Enzmann et al. 2018; Macrez et al. 2011; Wu et al. 2020). Among these processes, the inflammatory response is the main cause of neuronal death and infarction after CI/RI (Lindsberg and Grau 2003). Therefore, effective control of the inflammatory response is important for reducing CI/RI.

Accumulating evidence shows that microglial activation plays a vital role in inflammation after cerebral ischemia (Ma et al. 2017). Microglia are activated and polarized toward the inflammatory or anti-inflammatory phenotype within minutes after brain ischemia. Inflammatory microglia, also known as M1 microglia, secrete proinflammatory factors [tumor necrosis factor-alpha (TNF-α), interleukin-1 beta (IL-1β), IL-6, IL-12, and IL-23] and induce neuronal damage (Gaire et al. 2018). Anti-inflammatory microglia are also known as M2 microglia or alternatively activated microglia. They produce anti-inflammatory cytokines [IL-4, IL-10, transforming growth factor-beta (TGF-β), and IL-13] and release a variety of neurotrophic factors [vascular endothelial growth factor (VEGF), brain-derived neurotrophic factor (BDNF), platelet-derived growth factor (PDGF)] to protect against neuronal damage (Franco and Fernandez-Suarez 2015; Zeng et al. 2018). Activation of nuclear factor kappa B (NF-κB) signaling is responsible for microglial M1 polarization, and cyclic adenosine monophosphate response element-binding protein, a transcriptional competitor of NF-κB, inhibits the activation of the NF-κB pathway and contributes to promoting microglial M2 polarization (Zhao et al. 2019). Therefore, regulating microglial polarization is an important strategy for suppressing cerebral ischemia-induced neuroinflammation.

The incidence of stroke is lower in women who have not reached menopause than in men but higher in women who have reached menopause than in men, suggesting that estrogen and its receptor may play important roles in preventing stroke. G protein-coupled estrogen receptor (GPER) is expressed at higher levels in the brain than classical estrogen receptors (McEwen and Milner 2017). It is involved in inhibiting microglial activation and promoting M2 polarization (Pan et al. 2018; Zhang et al. 2018). Multiple studies have suggested that either endogenous or exogenous estrogen alleviate inflammatory injury caused by cerebral ischemia through GPER (Luo et al. 2018b; Thakkar et al. 2018). Icaritin (ICT), a phytoestrogen, is extracted from the dried stems and leaves of *Epimedii*. A phase I/II clinical trial of ICT for hepatocellular carcinoma has been completed, and two phase III studies are currently underway in China (Fan et al. 2019; Qin et al. 2020; Sun and Qin 2021a, b). ICT activates the extracellular signal-regulated kinase (ERK) signaling pathway, promotes the proliferation of bone mesenchymal stem cells, and enhances the differentiation and migration of osteoblasts when the estrogen receptor is activated (Luo et al. 2018a; Wu et al. 2017). Evidence also suggests that ICT relieves lipopolysaccharide-induced neuroinflammation in the hippocampus by inhibiting the NF-κB signaling pathway (Liu et al. 2019a). In the present study, we investigated the role of ICT in regulating microglial M1/M2 polarization after CI/RI and the related mechanism.

## Materials and methods

### Animals

All animal procedures were performed according to the China Council on Animal Care guidelines and were approved by the Gannan Medical Ethics Committee (Ganzhou China). Adult male specific pathogen-free SD rats (Hunan Slake Jingda Company, animal license number: SCXK (Xiang) 2019-0004) weighing 260–280 g were housed in a temperature-controlled (23 ± 2 °C) room with a relative humidity of 55 ± 15% on a 12 h light/dark cycle with free access to food and water.

### Therapeutic agents and reagents

ICT (C_21_H_20_O_6_, MW = 368.38 g/mol, purity ≥ 99%) was supplied by Shanghai Tianshui Chemical Co., Ltd., (Shanghai, China) and was dissolved in dimethyl sulfoxide (DMSO, D2650, Sigma, Saint Louis, MO USA). U0126 (S1102, Selleckchem, Germany) was dissolved in DMSO. Primary antibodies against Iba1 (ab5076), CD40 (ab13545), CD68 (ab125212), CD206 (ab125028), and GPER (ab39742) were purchased from Abcam (Cambridge, UK). Primary antibodies against P65-NF-κB (8242s), p-P65-NF-κB (3033s), ERK (4695s), and p-ERK (4370s) were purchased from Cell Signaling Technology (Danvers, MA, USA). β-Tubulin (MA5-11732) and β-actin (MA515739) antibodies were obtained from Invitrogen (California, USA). Rat IL-1β, IL-10 and TNF-α enzyme-linked immunosorbent assay (ELISA) kits were purchased from R&D Systems (Minnesota, USA). 2,3,5-Triphenyltetrazolium chloride (TTC) was obtained from Sigma (Darmstadt, Germany), high-efficiency RIPA lysis buffer and PMSF were obtained from Solarbio (Beijing, China), and a Fluoro-Jade C (FJC) Ready-to-Dilute Staining Kit was purchased from Biosensis (Thebarton, Australia).

### Transient middle cerebral artery occlusion (tMCAO) model

The rat tMCAO model was constructed using the modified Zea Longa suture method as described previously (Li et al. 2017; Liu et al. 2017, 2021; Shan et al. 2021). Rats were anesthetized by administering an intraperitoneal injection of 1% pentobarbital sodium at a dose of 4.5 mL/kg. An incision was made in the middle of the neck to expose the right common carotid artery (CCA), external carotid artery (ECA) and internal carotid artery (ICA). A small incision was made in the wall of the right ECA, and a nylon thread (A5-263620, Beijing Cinontech Co., Ltd., China) was inserted and slowly pushed into the ICA through the bifurcation of the CCA until it reached the opening of the middle cerebral artery. Two hours later, the nylon thread was pulled out through the ECA to allow reperfusion. The sham group rats underwent a similar operation without occlusion of the middle cerebral artery. The room temperature was strictly maintained at 23–25 °C during the operation.

### Lateral ventricle injection

Lateral ventricle injections were performed according to the experimental procedure reported in our previous studies (Xie et al. 2021). After being anesthetized, the rats were fixed on a stereotaxic apparatus, and the skin and subcutaneous tissues were separated to expose the skull and the anterior fontanelle. A cranial drill was used to drill holes (1.6 mm lateral and 0.9 mm posterior to bregma). Then, an injector was introduced through the bore to a subdural depth of 3.5 mm, and U0126, G15, or DMSO was injected into the lateral ventricle at a rate of 0.25 μL/min. The injection needle was retained in place for 5 min after administration to prevent backflow.

### Experimental groups

The experiment was mainly divided into two parts. In part I, rats were randomly divided into four groups: the sham group, ICT group, tMCAO group and tMCAO + ICT group. After ischemia for 10 min, rats in the ICT group and the tMCAO + ICT group were treated with ICT (0.5 mg/kg) by i.p. injection, whereas rats in the sham group and the tMCAO group were treated with the same volume of DMSO. In part II, rats were randomly divided into three groups to verify the inhibitory effects of GPER and ERK on ICT-mediated neuroprotection: the vehicle group, U0126 group and G15 group. The three groups of rats were injected with 1 μL of DMSO, U0126 (1 μg/μL) or G15 (25 μg/μL) into the lateral ventricle.

### Tests of neurological function

After 24 h of reperfusion, the degree of neurological impairment was assessed using the Garcia scale, which evaluates spontaneous activity, symmetry in the movement of the four limbs, forepaw outstretching, climbing, body proprioception and response to vibrissae touch (Garcia et al. 1995). The highest score (18 points) indicates normal function, and the lowest score (3 points) indicates the most severe functional impairment, as shown in Table 1.

**Table 1:** Garcia JH Neurological Scoring Criteria for rats

Test	0	1	2	3
Spontaneous activity (The rat was observed for 5 min in cage)	No movement	Barely moves	Move and touching at least one side of cage	Moving and touching at least three side of cage
Limb symmetry (Observation of four limbs after rats suspended)	Left limb: no movement	Left limb: slight movement	Left limb: slowness of movements	Symmetrical extension of limbs
Symmetry of forelimbs (Lift the tail to observe forelimb movement)	Left forelimb: no movement, no outreaching	Left forelimb: slight movement to outreach	Left forelimb: moves and outstretches less than the right	Symmetry outstretch
Climbing (45° of wire climbing)	–	Fails to climb	Left side is weak	Climb normally, hold tight to the wire
Body proprioception	–	Left unresponsive	The left stimulus response was slow than that right	Both side reactions are the same
Response to vibrissae touch	–	Left unresponsive	The left stimulus response was slow than that right	Both side reactions are the same

### Rotarod test

A rotarod (Jinan Yiyan Technology Development Co., Ltd., China) consisted of a rotating cylinder of approximately 8.5 cm in diameter and was used to evaluate the motor coordination of rats. The rats were required to continuously walk forward to stop themselves from falling off the rotating cylinder. The rats were trained twice daily for 2 days prior to tMCAO surgery. In the formal experiment, which was conducted 24 h after surgery, the rats were placed on the rotating rod, and the speed of the rod was increased from 4 to 40 rpm within 6 min (Doeppner et al. 2014). The test was performed twice, and the mean values were used for statistical analysis.

### TTC staining and measurement of the infarct volume

After the behavioral test, the rats were anesthetized and decapitated. Their brains were removed and cut into 5 2-mm-thick coronal sections. The brain slices were stained with 0.5% TTC in a 37 °C water bath for 15 min in the dark, washed with PBS, and then fixed with 4% paraformaldehyde (PFA) for 4–6 h. The slices were scanned with a scanner, and the brain infarct area was analyzed with ImageJ software (National Institutes of Health, Bethesda, MD, USA). The percentage of cerebral infarct volume (%) was calculated as (contralateral hemisphere volume − noninfarct ipsilateral hemisphere volume)/(2 × contralateral hemisphere volume) × 100 (Liu et al. 2021).

### Brain water content measurement

After being anesthetized, the rats were quickly decapitated, and whole brain tissues were removed. The water on the surface of the tissue was absorbed with filter paper. The right hemisphere was weighed to obtain the wet weight (W), and the left hemisphere was placed in a 60 °C oven for 72 h and then weighed to obtain the dry weight (D). Brain water content (%) was calculated as (W − D)/W × 100.

### ELISA

Rat brain tissue was collected from the ischemic penumbra for homogenization, and the supernatant was collected after centrifugation. The levels of IL-1β, IL-10, and TNF-α were determined using commercially available ELISA kits according to the manufacturer’s protocols.

### Quantitative PCR (qPCR)

The mRNA levels of CD11b, CD206, CD68, IL-1β, TNF-α and NF-κB p65 in the rat cerebral ischemic penumbra were measured using qPCR. Total RNA was extracted from the brain tissue using a TRIzol RNA Mini Kit (Ambion) according to the manufacturer’s instructions. Total RNA (3 μg) was transcribed into cDNAs using a Reverse Transcription Master Mix Kit (Invitrogen). qPCR was performed using EvaGreen and the BioMark HD Nanofluidic qPCR System combined with the GE 96.96 Dynamic Array IFC System. The relative expression levels of the genes were calculated using the 2^−ΔΔCT^ method by comparing the expression of the gene to that of β-actin, an endogenous control gene. The primers (5′-3′) used in this study are listed in Table 2. Experiments were performed in triplicate.

**Table 2:** The information of primers used in this study

Gene name	Primer sequences	
	Forward	Reverse
ACTB	CTAAGGCCAACCGTGAAAAG	ACCAGAGGCATACAGGGACA
CD11b	CTGCTCCTCAAGGTCGTTGT	AGATGGCGTACTTCACAGGC
CD206	TTCCTTTGGACAGACGGACG	TCCCTGCCTCTCGTGAATTG
CD68	TTCGGGCCATGCTTCTCTTG	GTCTCCGGGTAACGCAGAAG
IL-1β	AACTCAACTGTGAAATGCCACC	CATCAGGACAGCCCAGGTC
NF-κB p65	GGACCTATGAGACCTTCAAGAG	CAGAAGTTGAGTTTCGGGTAGG
TNF-α	CCACCACGCTCTTCTGTCTAC	AGGGTCTGGGCCATAGAACT

### Western blot

After 24 h of reperfusion, rat brain tissue from the ischemic penumbra was collected and lysed in high-efficiency RIPA lysis buffer containing PMSF. The protein concentration was determined using a BCA assay. Equal amounts of protein were loaded and separated on 10–12% sodium dodecyl sulfate-polyacrylamide gels and then transferred onto polyvinylidene difluoride (PVDF) membranes in Tris-glycine transfer buffer. The membranes were blocked with 5% skim milk for 1 h at room temperature and incubated with a primary antibody against β-actin (1:1000), β-tubulin (1:1000), Iba1 (1:1000), CD40 (1:1000), CD68 (1:1000), CD206 (1:1000), GPER (1:1000), P65-NF-κB (1:1000), p-P65-NF-κB (1:1000), ERK (1:1000), or p-ERK (1:1000) at 4 °C overnight. The membranes were then incubated with a goat anti-mouse secondary antibody (1:5000; LI-COR, USA) or goat anti-rabbit secondary antibody (1:5000; LI-COR, USA) at room temperature for 1 h. The bands corresponding to the antigen–antibody complexes were detected using Super Signal West Pico Chemiluminescent Substrate and visualized with an Amersham Imager 600 system.

### Immunofluorescence staining

After being anesthetized, the rats were transcardially perfused with PBS and 4% PFA for internal fixation. Then, the whole brain was removed, placed in PFA for external fixation for 24 h, and subjected to gradient dehydration with 20% and 30% sucrose. The brain tissue was cut into sections (30 μm) using a cryomicrotome and then incubated with 3% BSA to block nonspecific antibody binding. The sections were incubated with the following primary antibodies overnight at 4 °C: Iba1 (1:300), CD206 (1:500), and CD68 (1:300). The sections were rinsed with PBS and incubated with appropriate secondary antibodies, including Alexa Fluor 546-conjugated donkey anti-rabbit IgG (H+L) (1:1000) and Alexa Fluor 488-conjugated donkey anti-goat IgG (H+L) (1:1000), for 1 h at room temperature. The sections were then washed with PBS and mounted on coverslips with an anti-fluorescence quenching agent containing DAPI. Images were captured with a Zeiss fluorescence microscope (Zeiss, Oberkochen, Germany) and analyzed using Zeiss ZEN software.

### FJC staining

Degenerating neurons were identified by performing FJC staining. A series of sections containing the ischemic penumbra were selected and stained with an FJC Ready-to-Dilute Staining Kit. The sections were photographed with a Zeiss fluorescence microscope at an excitation wavelength of 488 nm. FJC-positive neurons were manually counted using ImageJ software (Gamdzyk et al. 2018).

### Statistical analysis

Statistical analyses of experimental data were performed using GraphPad Prism 8.0 software. Quantitative data are shown as the means ± standard deviations. Comparisons among groups were performed using one-way ANOVA, and pairwise comparisons were performed using Tukey’s multiple-comparison test. The significance threshold was set at *P* < 0.05.

## Result

### Neuroprotective effects of ICT on tMCAO rats

**Fig. 1:**
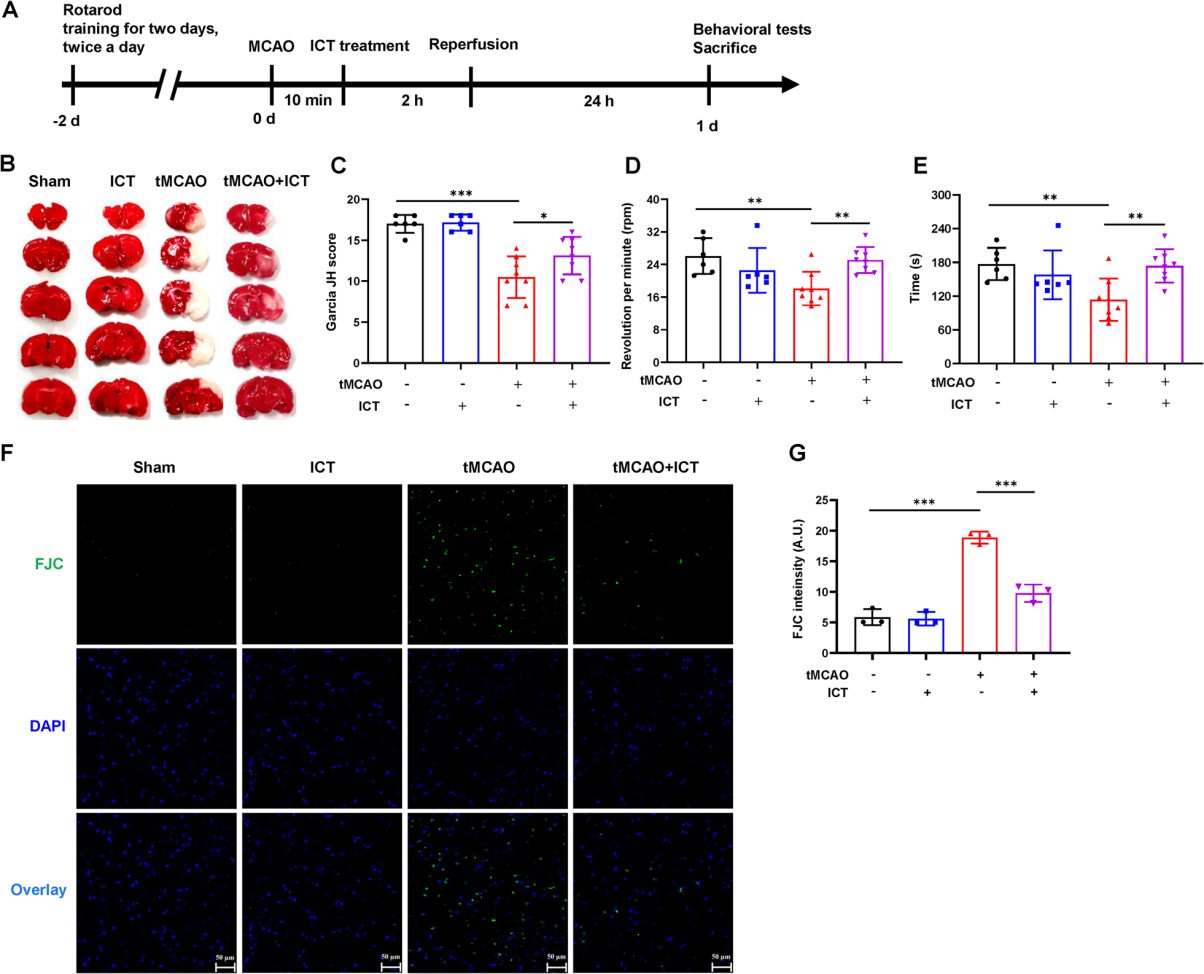
The protective effects of ICT on the cerebral ischemia rat. **A** The experimental timeline. **B** TTC-stained brain sections in the different groups. **C** Neurological function scores (n = 6–8). **D** The rotating speed in rotarod test. **E** The time on rod in rotarod test (n = 6–8). **F** and **G** Degenerating neurons were measured by FJC staining. Green fluorescence showed degenerating neurons, and blue fluorescence represented DAPI (n = 3), scale bar = 50 μm. The results are expressed as the mean ± SD, *P < 0.05, ***P < 0.001

First, we evaluated the therapeutic effect of ICT on tMCAO rats through TTC staining, neurological function assessments and the rotarod test (Fig. 1A). TTC stained the normal nonischemic brain tissue red, whereas the infarcted area did not retain the dye and therefore appeared white. The infarct volume in the cerebral cortex was markedly smaller in the tMCAO + ICT group than in the tMCAO group (Fig. 1B). We used a double-blind method to evaluate neurological function with the Garcia scale. The neurological score was significantly reduced in the tMCAO group but increased after ICT treatment (Fig. 1C). Rats performed the rotarod test to evaluate the locomotor function of tMCAO rats. The latency to fall was significantly shorter and the rotation speed was significantly slower in the tMCAO group than in the sham group, and these parameters were significantly increased after ICT treatment (Fig. 1D, E).

Neuronal degeneration occurs in the early stage of stroke. We evaluated the effect of ICT on neuronal degeneration in the ischemic penumbra of tMCAO rats using FJC staining. The number of FJC-positive cells and the FJC fluorescence intensity were significantly increased in the tMCAO group compared with the sham group and were significantly decreased after ICT treatment (Fig. 1F, G). The results suggested that ICT exerted protective effects on neuronal injury in tMCAO rats.

### ICT relieved neuroinflammation in tMCAO rats

**Fig. 2:**
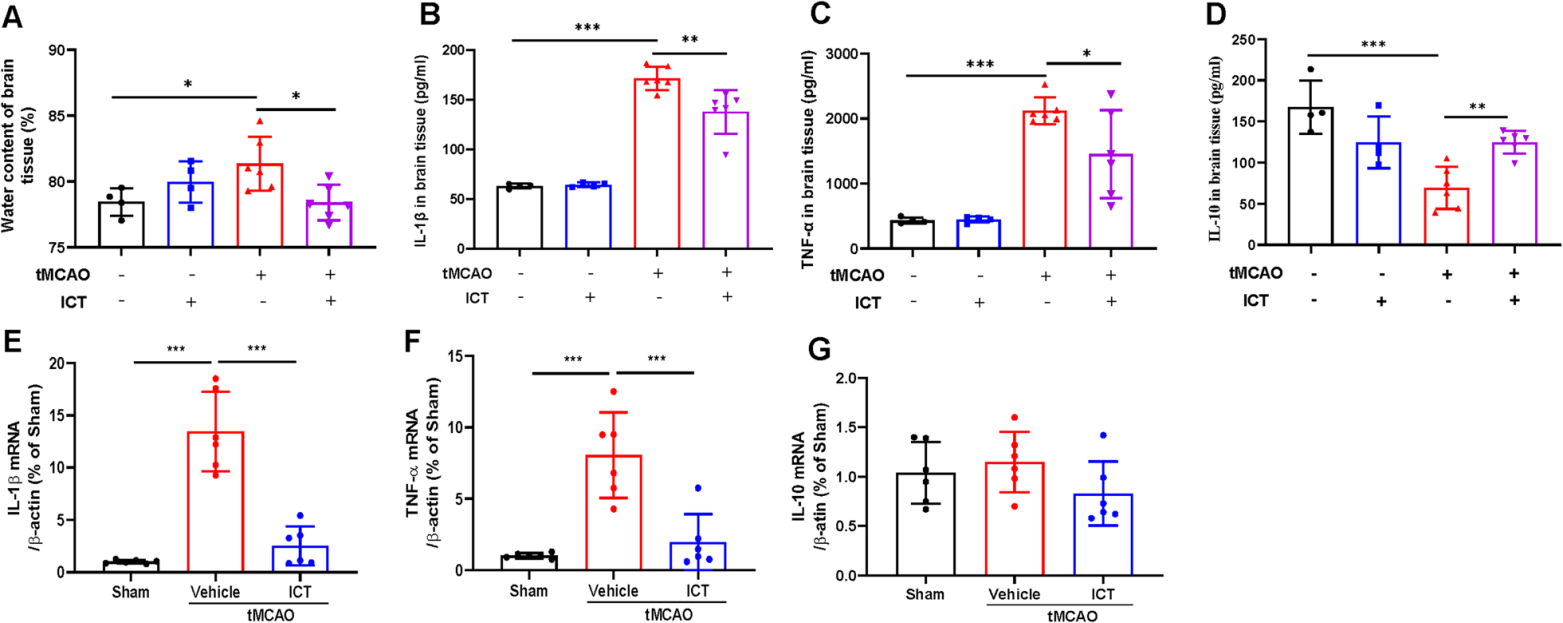
ICT attenuates brain edema and neuroinflammation in rats after CI/RI. **A** The water content of rat ischemic lateral brain tissue (n = 4–6). **B**–**D** IL-1β, TNF-αand IL-10 in brain tissue of rat ischemic penumbra (n = 4–6). **E**–**G** The mRNA expression of IL-1β, TNF-α and IL-10 in the ischemic penumbra (n = 6). The results are expressed as the mean ± SD, *P < 0.05, **P < 0.01, ***P < 0.001

We measured the water content and the levels of inflammatory factors in the ischemic penumbra to assess the effects of ICT on cerebral edema and inflammation in tMCAO rats. The water content was significantly increased in the tMCAO group compared with the sham group following 24 h of reperfusion, and this change was alleviated by ICT treatment (Fig. 2A). The levels of the proinflammatory factors IL-1β and TNF-α in the ischemic penumbra were significantly increased, and the level of the anti-inflammatory factor IL-10 was significantly decreased. After ICT treatment, the levels of IL-1β and TNF-α were significantly decreased, and that of IL-10 was significantly increased (Fig. 2B–D). qPCR further confirmed that ICT treatment reduced IL-1β and TNF-α mRNA expression in the ischemic penumbra (Fig. 2E, F). We also examined IL-10 mRNA expression, but it did not change (Fig. 2G). Based on these results, ICT ameliorated cerebral edema and neuroinflammation in rats after CI/RI.

### The effect of ICT on microglial activation and polarization in tMCAO rats

**Fig. 3:**
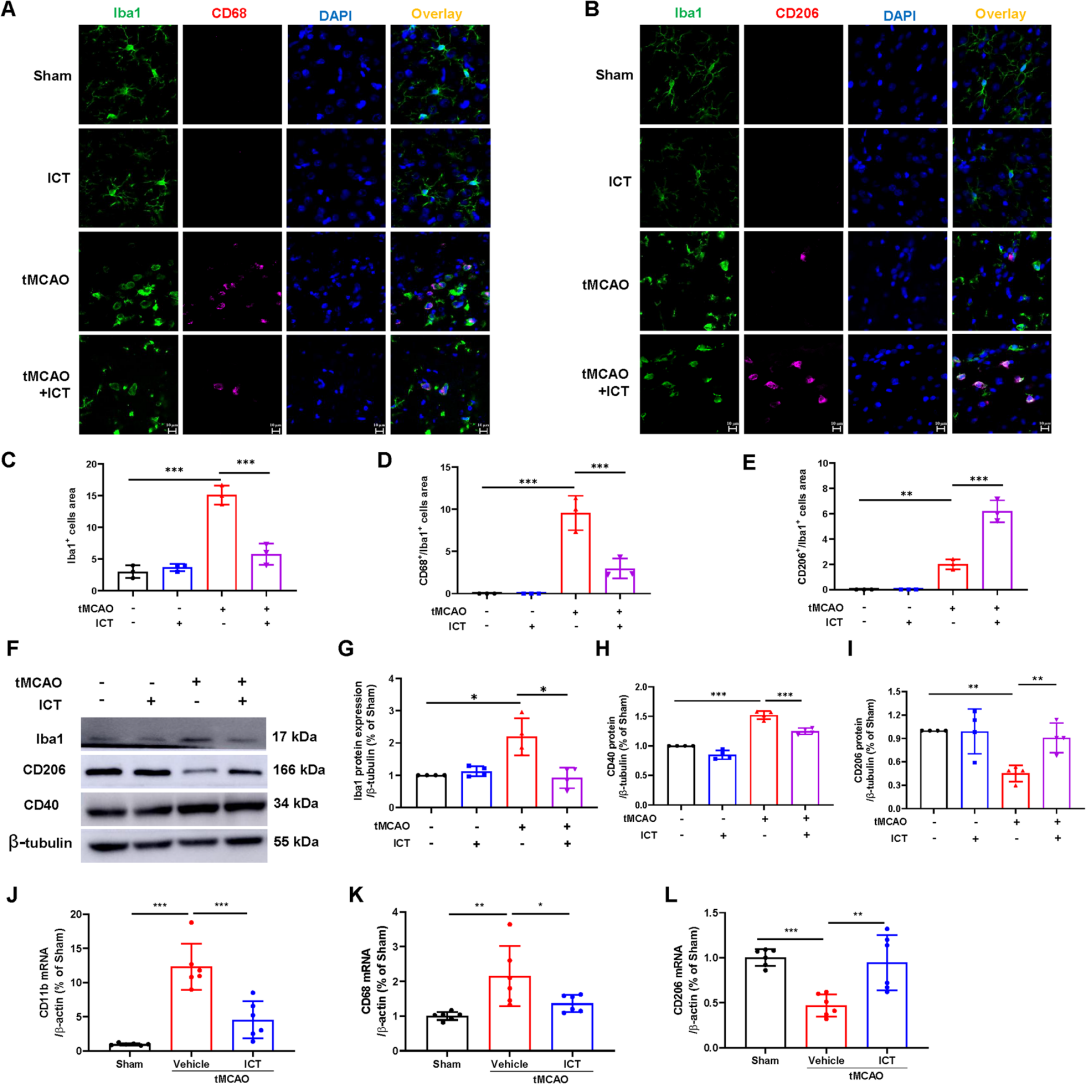
The effect of ICT on activation and polarization of microglia in tMCAO rats. **A** and **B** Representative immunofluorescent images of microglia polarization in the ischemic penumbra. The immunofluorescence staining of microglia with anti-CD68 (magenta), CD206 (magenta), and Iba1 (green) antibody. The nucleus was stained by DAPI; scale bar = 10 μm. **C**–**E** Immunofluorescence quantitation (n = 3). **F**–**I** The protein expressions of Iba1, CD40 and CD206 (n = 4). **J**–**L** The mRNA expression of CD11b, CD68, and CD206 (n = 6). The results are expressed as the mean ± SD, *P < 0.05, **P < 0.01, ***P < 0.001

We examined the expression of Iba1, an important marker of microglial activation, in the ischemic penumbra of rats to explore whether ICT treatment reduced neuroinflammation by affecting microglia. The number of activated microglia and Iba1 protein expression levels were significantly increased in the tMCAO group but significantly decreased after ICT treatment (Fig. 3A–C, F, G). In addition, CD11b mRNA expression showed a consistent change (Fig. 3J).

We explored the effect of ICT on microglial polarization by performing immunofluorescence staining, western blotting and qPCR to measure the expression of M1 and M2 markers (CD68 and CD40 for M1 and CD206 for M2 polarization). Our results showed that CI/RI, which typically promotes M1 polarization, upregulated the expression of CD68 and downregulated the expression of CD206. After ICT treatment, CD68 and CD40 expression were significantly increased, and CD206 expression was decreased (Fig. 3A–I). ICT treatment also downregulated CD68 mRNA expression and upregulated CD206 mRNA expression (Fig. 3K–L).

### ICT regulated the activation of the ERK-NF-κB pathway in tMCAO rats

**Fig. 4:**
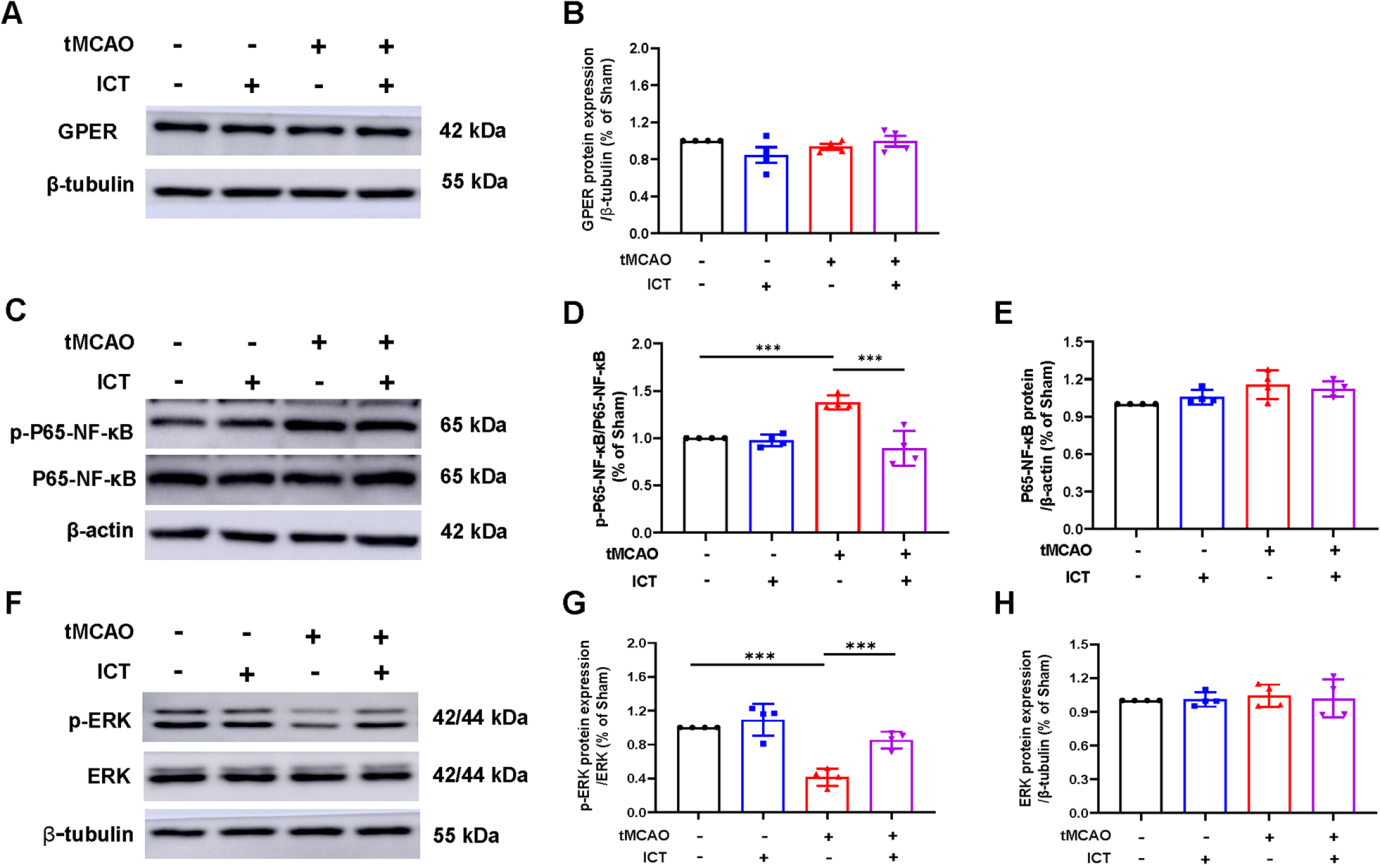
The expression levels of GPER, P65-NF-κB, ERK and the phosphorylation level of P65-NFκB, ERK are regulated by ICT treatment after CI/RI. **A**–**H** The protein expression of GPER, p-P65-NF-κB, P65-NF-κB, ERK and p-ERK (n = 4). The results are expressed as the mean ± SD, *P < 0.05, **P < 0.01

Western blotting was used to measure the levels of GPER, p-P65-NF-κB and p-ERK in the ischemic penumbra and assess the effect of ICT on the GPER–ERK–NF-κB signaling pathway. We did not observe differences in GPER expression levels among the groups (Fig. 4A, B). p-P65-NF-κB levels were significantly increased and p-ERK levels were decreased in the tMCAO group compared with the sham group. Levels of p-P65-NF-κB were reduced and p-ERK levels were increased in the tMCAO + ICT group compared with the tMCAO group. No changes in total P65-NF-κB and ERK levels were observed among the groups (Fig. 4C–H).

### G15 and U0126 reversed the neuroprotective effects of ICT on tMCAO rats

**Fig. 5:**
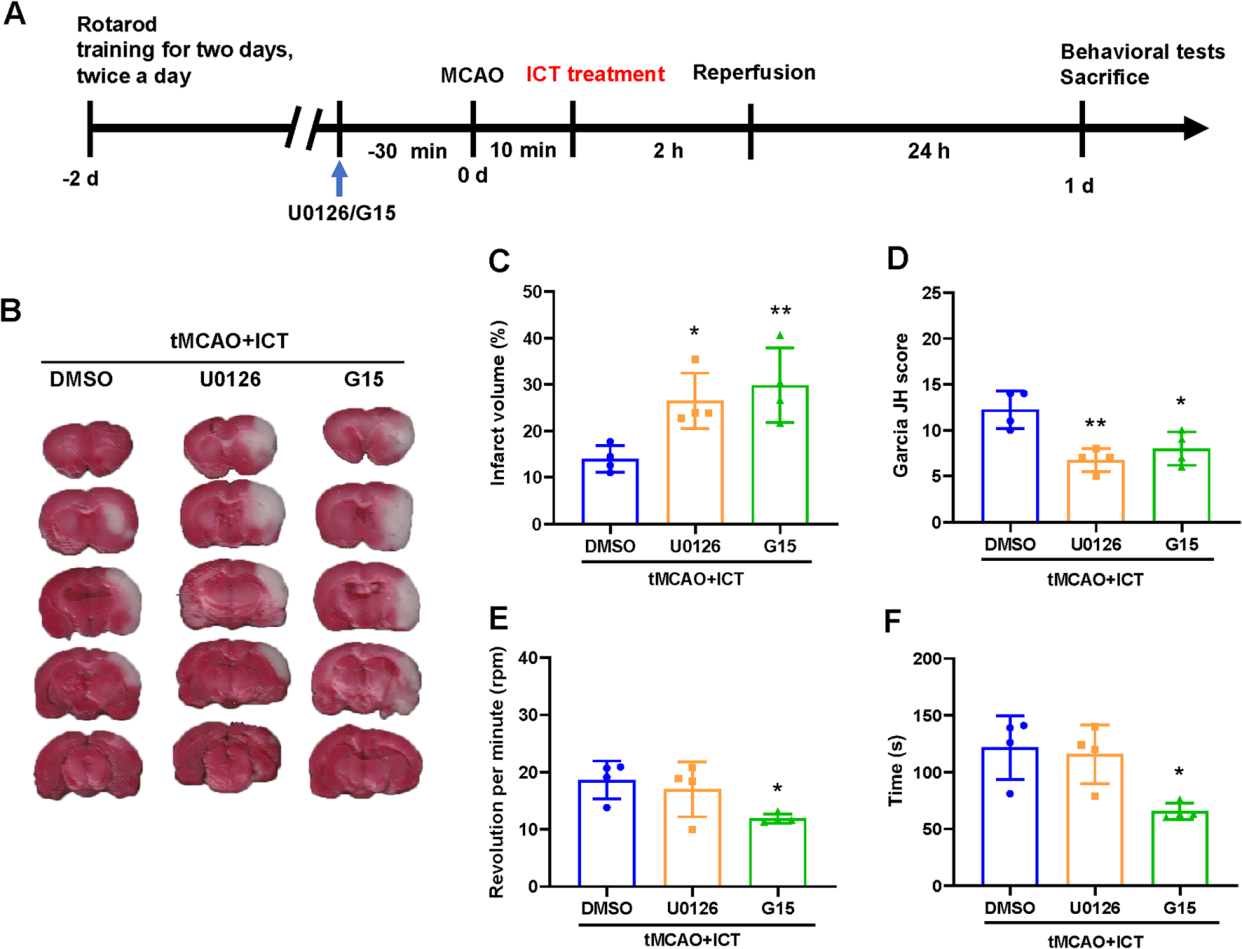
G15 and U0126 reversed the neuroprotective effects of ICT in tMCAO rats. **A** The experimental timeline. **B** and **C** The cerebral infarct volume after tMCAO in brain tissue in the different groups were analyzed by TTC staining (n = 4). **D** Neurological function scores. **E** The rotating speed in rotarod test (n = 4). **F** The time on rod in rotarod test (n = 4). The results are expressed as the mean ± SD, *P < 0.05, **P < 0.01, compared with tMCAO + ICT group

We administered G15 (a GPER inhibitor) or U0126 (an ERK inhibitor) and evaluated the effects of ICT on tMCAO rats to further verify whether ICT regulated microglial activation and polarization by modulating the activity of the GPER–ERK–NF-κB signaling pathway (Fig. 5A). The infarct volume was significantly increased and the Garcia score, latency to fall and rotation speed were decreased in the G15- and U0126-treated groups compared with the vehicle group (Fig. 5B–F). Thus, the neuroprotective effect of ICT on tMCAO rats was reversed by the two inhibitors, suggesting that the effect of ICT on ischemic stroke may be achieved via the activation of GPER–ERK signaling.

### G15 and U0126 reversed the anti-inflammatory effect of ICT on tMCAO rats

**Fig. 6:**
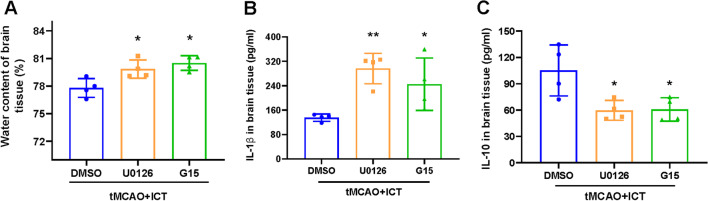
U0126 and G15 reversed the effect of ICT on reducing brain edema and neuroinflammation in tMCAO rats. **A** The water content of rat ischemic lateral brain tissue (n = 4). **B** and** C** IL-1β and IL-10 levels in rat brain tissue in ischemic penumbra area (n = 4). The results are expressed as the mean ± SD, *P < 0.05, **P < 0.01, compared with tMCAO + ICT group

After the administration of G15 or U0126, the water content and IL-1β level in the ischemic penumbra were significantly increased, and the IL-10 concentration was significantly decreased in the G15- and U0126-treated groups compared with the vehicle group (Fig. 6A–C). These findings showed that the two inhibitors reversed the inhibitory effect of ICT on neuroinflammation, suggesting that the anti-inflammatory effect of ICT may be achieved via the activation of GPER–ERK signaling.

### G15 and U0126 reversed the effect of ICT on the activation and polarization of microglia in tMCAO rats

**Fig. 7:**
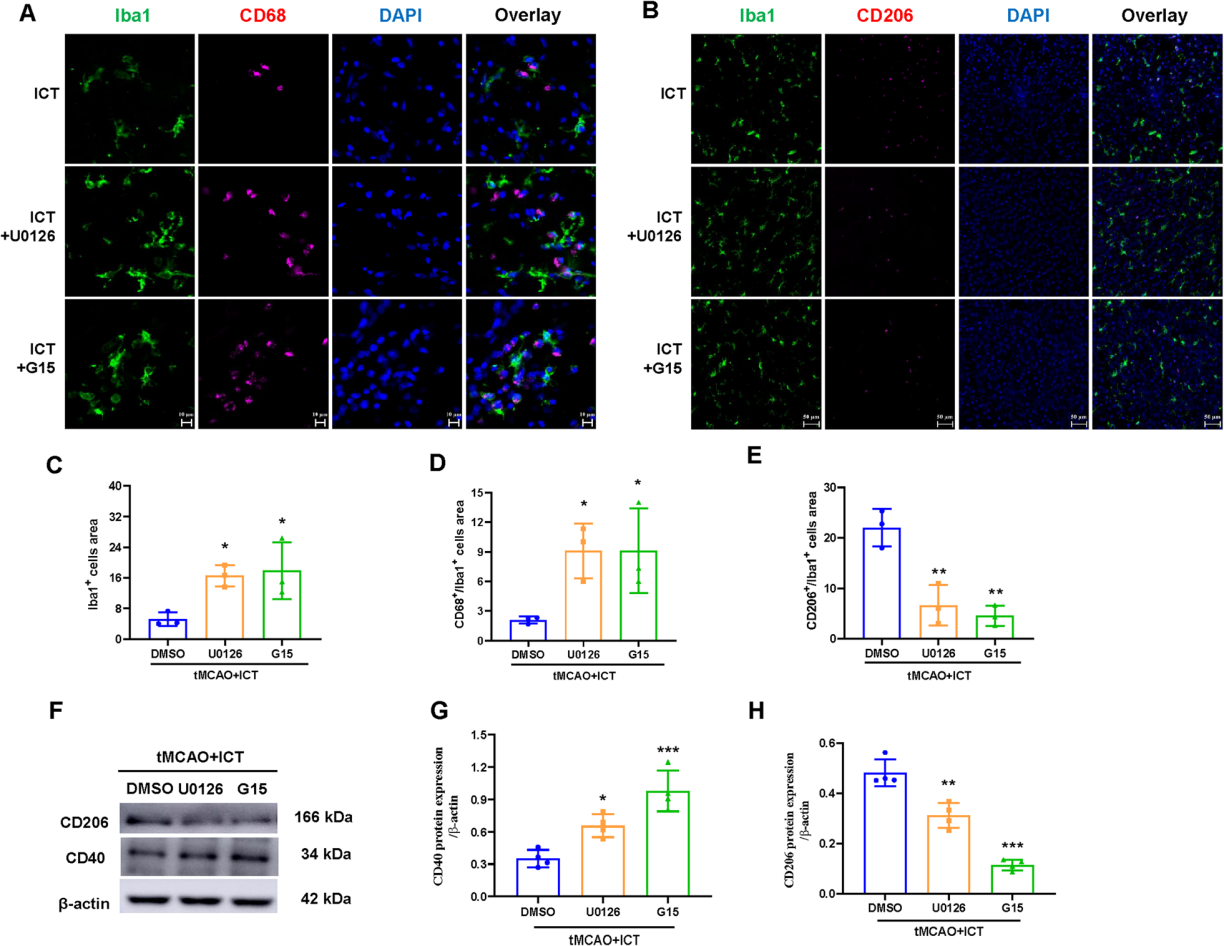
U0126 and G15 reversed the effect of ICT on the phenotypic polarization of microglia M1/M2 in tMCAO rats. **A** and **B** Representative immunofluorescent images of microglia polarization in the ischemic penumbra. The immunofluorescence staining of microglia with anti-CD68 (magenta), CD206 (magenta), and Iba1 (green) antibody. The nucleus was stained by DAPI; **A**: scale bar = 10 μm; **B**: scale bar = 50 μm. **C**–**E** Immunofluorescence quantitation (n = 3). **F**–**H** The protein expressions of CD40 and CD206 (n = 4). The results are expressed as the mean ± SD, *P < 0.05, **P < 0.01, ****P < 0.0001, compared with tMCAO + ICT group

We measured the expression of M1 and M2 markers using immunofluorescence staining and western blotting to confirm our hypothesis. G15 or U0126 treatment increased the expression of Iba1, CD68 and CD40 and decreased the expression of CD206 (Fig. 7A–H). Based on these results, the two inhibitors reversed the regulatory effect of ICT on microglial activation and polarization, indicating that GPER–ERK signaling was involved in the effects of ICT on tMCAO rats.

### G15 and U0126 reversed the effect of ICT on the activation of ERK and NF-κB signaling in tMCAO rats

**Fig. 8:**
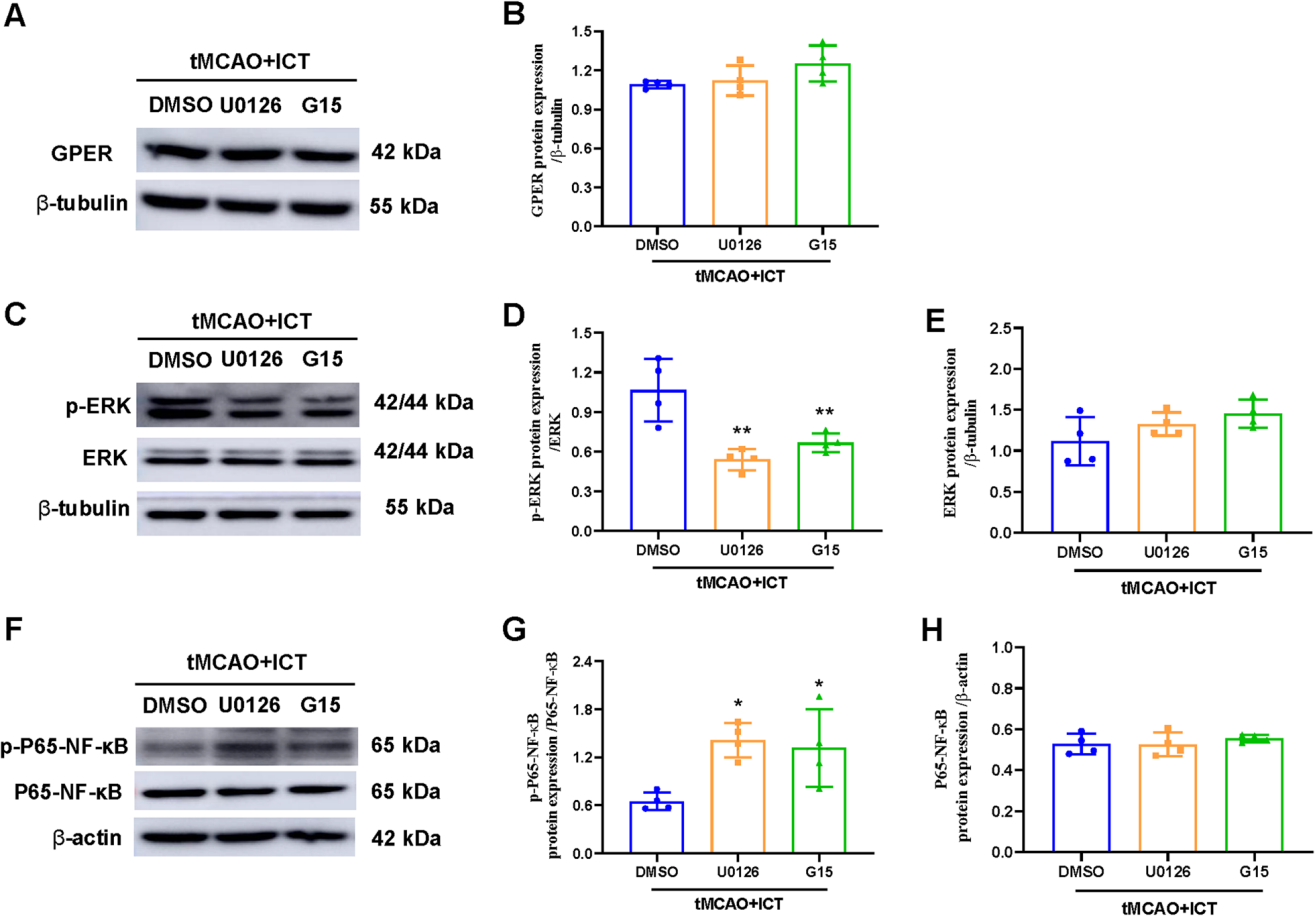
U0126 and G16 reversed effects of ICT on ERK-NF-κB signaling in CI/RI rats. **A**–**H** The protein expression of GPER, p-P65-NF-κB, P65-NF-κB, ERK and p-ERK (n = 4). The results are expressed as the mean ± SD, *P < 0.05, **P < 0.01, compared with tMCAO + ICT group

U0126 and G15 did not alter the expression of the GPER protein in ICT-treated tMCAO rats (Fig. 8A). After the administration of G15 or U0126, the phosphorylation of the ERK protein in the ischemic penumbra of ICT-treated tMCAO rats was decreased, while total ERK protein expression was not affected (Fig. 8C–E). After the administration of G15 or U0126, the phosphorylation level of the NF-κB protein in the ischemic penumbra of ICT-treated tMCAO rats was increased, while total NF-κB protein expression was not affected (Fig. 8F–H). These results suggested that G15 reversed the ICT-induced increase in p-ERK levels and reduction in p-P65-NF-κB levels and that U0126 reversed the ICT-induced reductions in p-P65-NF-κB levels. Therefore, ICT might activate ERK through GPER and then inhibit the activation of NF-κB in the ischemic penumbra of rats.

## Discussion

Ischemic stroke is a sudden, acute brain disease. At present, the only therapeutic drug for ischemic stroke is tissue-type plasminogen activator, but its therapeutic time window is only 4.5 h (Nogueira et al. 2018). ICT has a low molecular weight and high lipophilicity, easily crosses the blood‒brain barrier and has a long half-life in the body (Huang et al. 2019). We found that ICT improved the neurological function of tMCAO rats and significantly reduced the number of degenerated neurons, proving that ICT exerts a neuroprotective effect on rats subjected to CI/RI.

Inflammation is now recognized as a principal factor in brain injury and neurological dysfunction after CI/RI. After acute cerebral ischemia, damaged neurons and glial cells secrete proinflammatory factors such as IL-1β and TNF-α, which increase the permeability of the blood‒brain barrier by directly affecting multiple capillaries, leading to cerebral edema and allowing peripheral immune cells and cytokines to enter brain tissue, further aggravating inflammation and brain injury (Fu et al. 2015). In a rat model of chronic cerebral ischemia, Radix Salviae Miltiorrhizae extract was found to decrease TNF-α and IL-6 levels, thereby inhibiting the inflammatory response (Zhang et al. 2013). ICT effectively inhibits the DNA-binding activity of P65-NF-κB and the expression of IL-1β and TNF-α in brain tissue in mice subjected to ischemia/reperfusion injury (Sun et al. 2018). In our study, ICT treatment decreased the expression of IL-1β and TNF-α while increasing the level of IL-10, indicating that ICT inhibited neuroinflammation and cerebral edema induced by tMCAO in rats by decreasing proinflammatory cytokine levels and increasing anti-inflammatory cytokine levels.

Microglia have been recognized as important contributors to the occurrence and development of neuroinflammation induced by ischemic stroke. Microglia are rapidly activated and polarized when cerebral ischemia occurs; M1 microglia release proinflammatory factors, whereas M2 microglia produce anti-inflammatory factors and function in tissue repair (Qiu et al. 2020; Thomas et al. 2017). As shown in the present study, ICT effectively inhibited the activation of microglia in tMCAO rats. Thus, we evaluated the effect of ICT on microglial M1/M2 polarization. ICT reduced the expression of M1 microglial markers in the ischemic penumbra of tMCAO rats and increased the expression of M2 microglial markers. Based on these results, the antineuroinflammatory effect of ICT after ischemic stroke may be achieved by restraining microglial M1 polarization and promoting microglial M2 polarization.

NF-κB is a key transcription factor regulating the inflammatory response. When NF-κB is activated, it is phosphorylated and translocates to the nucleus, where it mediates inflammatory brain injury by inducing the transcription of many proinflammatory genes, such as IL-1β and TNF-α (Zhang et al. 2019). In vivo, genistein-3′-sodium sulfonate, which contains a structural modification of the phytoestrogen genistein, was shown to inhibit the activation and nuclear translocation of NF-κB and reduce the M1 polarization of microglia, thereby inhibiting neuroinflammation in tMCAO rats (Liu et al. 2021). Multiple in vitro studies have also revealed that NF-κB is involved in microglial activation and M1 polarization (Kong et al. 2022; Liu et al. 2019b). Interfering with the activation of NF-κB inhibits M1 polarization and reduces neuroinflammation induced by ischemia (Su et al. 2022). Our results suggested that ICT treatment reduced NF-κB activation in tMCAO rats, indicating that it is involved in regulating microglial polarization and inhibiting neuroinflammation induced by ICT.

Activation of ERK signaling reduces the lipopolysaccharide-induced nuclear translocation of NF-κB in BV2 microglia, thereby inhibiting M1 polarization (Mohanraj et al. 2019; Ni et al. 2015). In addition, ERK signaling may also promote the polarization of microglia toward the M2 phenotype (Wang et al. 2018). In our study, ICT treatment increased ERK activation, indicating that ICT may exert a regulatory effect on microglial polarization through ERK-mediated inhibition of NF-κB.

GPER is an estrogen-specific membrane receptor that exerts the rapid nongenomic effects of estrogen (Guan et al. 2017). In ovariectomized female and male rats, the GPER agonist G1 inhibits the upregulation of proinflammatory cytokines (IL-1β, IL-6, and TNF-α), increases the expression of the anti-inflammatory cytokine IL-4, and induces the polarization of microglia toward the M2 phenotype, thereby relieving cerebral ischemic injury (Lu et al. 2016). Therefore, GPER is an important target for the regulation of microglial polarization, inhibition of neuroinflammation and neuroprotection. We did not observe a change in GPER protein expression in rats after 24 h of ischemia/reperfusion, and ICT, U0126 and G1 did not alter GPER expression, consistent with an existing report (Lu and Herndon 2017). Our study also documented that the administration of a GPER or ERK inhibitor reversed the effects of ICT on rats with ischemic stroke, including its neuroprotective and antineuroinflammatory effects and its abilities to regulate the activation and polarization of microglia and inhibit NF-κB. Thus, these effects of ICT were achieved via the activation of GPER and ERK. In addition, the GPER inhibitor influenced ERK activation, but the ERK inhibitor did not regulate GPER expression, indicating that ERK may be one of the downstream signaling molecules of GPER. This finding suggested that ICT might regulate the polarization of microglia through the GPER–ERK–NF-κB pathway, thereby exerting a neuroprotective effect by reducing neuroinflammation in rats with ischemic stroke.

However, this study has some limitations. First, we only studied the protective mechanism of ICT, a phytoestrogen, on cerebral ischemia in male rats to exclude the effect of endogenous estrogen. A study has shown that the pattern of GPER expression is similar in the adult male and female rat brains (Brailoiu et al. 2007), and we speculate that ICT might also protect the brain of female rats with cerebral ischemia by activating GPER. Second, we did not assess the long-term outcomes of the rats, including the survival rate and mortality rate. In future studies, we will extend the observation period. Finally, we only performed prophylactic administration for 10 min after insertion of the nylon thread. However, the development of drugs for the treatment of stroke involves the problem of a therapeutic time window. Next, we will further explore whether the therapeutic time window for ICT is wider than that for tissue-type plasminogen activator.

## Conclusions

In summary, we investigated the protective effects of ICT on tMCAO rats and the related mechanisms. The findings of the present study are listed below. (1) ICT attenuates neuroinflammation, improves motor function, and repairs tissue after CI/RI in rats. (2) ICT regulates microglial activation and polarization to inhibit the inflammatory response. (3) ICT may regulate microglial activation and polarization through the GPER–ERK–NF-κB signaling pathway.

## Data Availability

The data that support the findings of this study are available from the corresponding author upon request. All of data were generated in-house, and no paper mill was used.

## Abbreviations

BDNF: Brain-derived neurotrophic factor
CCA: Common carotid artery
CI/RI: Cerebral ischemia reperfusion injury
DMSO: Dimethyl sulfoxide
ECA: External carotid artery
ERK: Extracellular signal regulated kinase
FJC: Fluoro-Jade C
GPER: G protein coupled estrogen receptor
ICA: Internal carotid artery
ICT: Icaritin
IL-1β: Interleukin-1 beta
NF-κB: Nuclear factor-kappaB
PDGF: Platelet derived growth factor
PFA: Paraformaldehyde
PVDF: Polyvinylidene difluoride
tMCAO: Transient middle cerebral artery occlusion
TGF-β: Transforming growth factor-beta
TNF-α: Tumor necrosis factor-alpha
TTC: 2,3,5-Triphenyltetrazolium chloride
VEGF: Vascular endothelial growth factor
